# Diagnostic Performance of Unattended Automated Office Blood Pressure Measurement for Hypertension Screening Among People With and Without HIV

**DOI:** 10.1161/JAHA.125.043957

**Published:** 2025-09-19

**Authors:** Ruth K. Lucinde, Megan Willkens, Benson Issarow, Salama Fadhil, Cody Cichowitz, Philip Ayieko, Godfrey Kisigo, Sara Venkatraman, Heiner Grosskurth, Ana C. Krieger, Richard B. Devereux, Myung Hee Lee, Saidi Kapiga, Robert N. Peck, Anthony O. Etyang

**Affiliations:** ^1^ Department of Epidemiology and Demography KEMRI‐Wellcome Trust Research Programme Kilifi Kenya; ^2^ Center for Global Health, Department of Medicine Weill Cornell Medicine New York NY USA; ^3^ Mwanza Intervention Trials Unit, National Institute of Medical Research Mwanza Tanzania; ^4^ Division of Cardiology, Department of Medicine University of California San Francisco San Francisco CA USA; ^5^ Department of Infectious Disease Epidemiology London School of Hygiene and Tropical Medicine London UK; ^6^ Weill Cornell Center for Sleep Medicine, Departments of Medicine Neurology and Genetic Medicine, Weill Cornell Medical College New York NY USA; ^7^ Division of Cardiology, Department of Medicine Weill Cornell Medicine New York NY USA; ^8^ Weill Bugando School of Medicine, Department of Medicine Catholic University of Health and Allied Sciences Mwanza Tanzania

**Keywords:** diagnostic performance, hypertension, people with HIV, unattended office blood pressure, Hypertension

## Abstract

**Background:**

The diagnostic performance of automated office blood pressure (AOBP) in screening for hypertension in people with HIV (PWH) is not known.

**Methods:**

We conducted a cross‐sectional analysis of baseline data from PWH and people without HIV (PWoH) from the Mwanza HIV&CVD cohort study. We conducted unattended AOBP and 24‐hour ambulatory BP monitoring as recommended by international guidelines. Using average 24‐hour BP as the reference standard, we estimated the prevalence of hypertensive diagnostic phenotypes and calculated measures of diagnostic performance at different diagnostic cutoffs in participants.

**Results:**

We included 959 participants (50.4% PWH and 49.6% PWoH). Characteristics were similar across participant groups. The median age was 44 years (interquartile range, 38–50 years), and 69.8% were women. Overall prevalence of hypertension, based on average 24‐hour ambulatory BP monitoring, was 35.3% using European Society of Hypertension cutoffs and did not differ by HIV infection status. Masked hypertension was present in 25.7% (95% CI, 22.0%–29.8%) of PWH and 26.7% (95% CI, 22.9%–30.8%) of PWoH. The sensitivity of unattended AOBP was 25.7% for PWH (95% CI, 19.3%–33.1%) and 25.7% for PWoH (95% CI, 19.4%–33.0%) with little difference in the area under the receiver‐operating curve by HIV infection status. Based on 24‐hour BP averages, 24.2% of PWH and 21.6% of PWoH had isolated nocturnal hypertension.

**Conclusions:**

More than half of individuals with hypertension on ambulatory BP monitoring, irrespective of their HIV infection status, may be misdiagnosed if unattended AOBP alone is used to screen for hypertension in sub‐Saharan Africa.

**Nonstandard Abbreviations and Acronyms**
ABPM: ambulatory blood pressure monitoring
ACC: American College of Cardiology
AHA: American Heart Association
ESH: European Society of Hypertension
PWH: people with HIV
PWoH: people without HIV
SSA: sub‐Saharan Africa
uAOBP: unattended automated office blood pressure

**Clinical Perspective**

**What Is New?**

- Unattended automated office blood pressure had low sensitivity in identifying individuals with hypertension when compared with average 24‐hour blood pressure for adults with and without HIV in Tanzania.
- The prevalence of masked hypertension and isolated nocturnal hypertension was high regardless of HIV status.

**What Are the Clinical Implications?**

- Reliance on automated office blood pressure measurements in sub‐Saharan Africa would fail to identify more than half of individuals who have hypertension on ambulatory monitoring.

Hypertension is a major risk factor for cardiovascular disease (CVD) and accounts for >20% of all deaths globally.^1^, ^2^ The region of sub‐Saharan Africa (SSA) has the highest age‐adjusted prevalence of hypertension^3^ and also the lowest rates of blood pressure (BP) treatment and control.^4^ There is an urgent need to improve hypertension diagnosis in Africa,^5^ but the best strategy for measuring BP in Africa has not yet been defined.

More than 25 million people in SSA are infected with HIV, comprising >65% of the global population of people with HIV (PWH). PWH experience a significantly higher risk for CVD.^3^, ^6^ This higher risk for CVD might be related to difference in BP phenotypes and pathophysiology between PWH and people without HIV (PWoH).^6^, ^7^ Improved screening and early diagnosis of hypertension will be critical for reducing CVD risk in PWH.^8^

Although screening and diagnosis of hypertension are typically done with single or multiple attended automated office BP (AOBP) measurements, unattended AOBP (uAOBP) measurements may reduce false positive diagnoses (white‐coat hypertension).^9^ However, the use of uAOBP in SSA is limited due to practical implementation challenges^10^ as well as lack of data on its diagnostic performance within this population, a population that exhibits a different pattern of hypertension compared with other regions.^11^, ^12^ Furthermore, higher rates of nondipping BP and nocturnal hypertension^13^, ^14^ among PWH may diminish the value of office BP measurements in general as they are unable to identify nocturnal hypertension.

Ambulatory BP monitoring (ABPM) is the reference standard BP measurement method for diagnosing hypertension both in the general population and in special populations like PWH.^15^, ^16^, ^17^ However, ABPM is not available in most health facilities in SSA. Therefore, the Pan‐African Society of Cardiology has suggested that repeated office BP measurements are the only “practical BP measurement” method in the region.^10^ Questions related to the diagnostic accuracy of uAOBP are particularly relevant to PWH in Africa given the recent push to integrate hypertension care with HIV care.^3^, ^18^ Therefore, we sought to determine uAOBP's diagnostic performance using 24‐hour ABPM as the reference standard and whether this performance differed between PWH and PWoH.

## METHODS

All data used during this study will be available from the corresponding author upon reasonable request.

### Study Design

This is a cross‐sectional analysis of data collected from the enrollment visit for a prospective cohort study being conducted in Mwanza, Tanzania. For our Mwanza HIV&CVD cohort, we recruited 500 PWH from 3 government HIV clinics in the city of Mwanza in northwest Tanzania. For comparison, 500 PWoH were chosen from the full set of treatment partners registered at these 3 clinics. In Tanzania, PWH are assigned treatment partners who support them through their HIV treatment journey. We have previously shown that treatment partners have similar characteristics to PWH attending the same clinics because they are drawn from the same source populations.^19^ All participants were adults aged >30 years.

### Data Collection

We collected sociodemographic data and conducted clinic uAOBP measurements and 24‐hour ABPM on all participants at baseline. Clinic uAOBP and 24‐hour ABPM were initiated on the same day. Clinic uAOBP was measured with the OMRON HBP‐1300 device (OMRON Healthcare, Kyoto, Japan^20^) following the procedures recommended by the joint American College of Cardiology/American Heart Association (ACC/AHA) guidelines for office/clinic BP measurements.^21^ Participants were allowed 5 minutes of rest in a quiet room at the study clinic before 3 automated BP measurements were conducted 30 seconds apart. No study staff were present in the room during the rest or measurement period. The average of the second and third measurements was used as the final recorded BP.

After completion of the uAOBP measurements, participants were fitted with an appropriately sized upper‐arm cuff on the nondominant arm and were sent home with an ambulatory device to collect 24‐hour ABPM measurements using the Mobil‐O‐Graph ABP system (IEM GmbH, Germany). Their BP was measured every 30 minutes during the day (6:00 a.m. to 10:00 p.m.) and every 60 minutes at night (10:00 p.m. to 6:00 a.m.). Participants were considered to have adequate 24‐hour ABPM measurements if they had at least 70% of their scheduled measurements conducted successfully including at least 3 valid asleep measurements. Sleep times during ABPM measurement were determined using self‐reported sleep diaries confirmed with 24‐hour actigraphy with the Micro Motionlogger Watch (Ambulatory Monitoring Inc, Ardsley, NY). These sleep times were used to define awake and asleep periods and to calculate mean awake and mean asleep BP. Mean 24‐hour BP was defined as the time‐weighted average of valid awake BP and asleep BP readings.

### Hypertensive Status

We determined participant hypertensive status based on uAOBP using 2 BP cutoffs; the European Society of Hypertension (ESH) recommended cutoffs (ie, average office systolic BP >140 mm Hg or average office diastolic BP ≥90 mm Hg)^22^ for the primary analyses and the ACC/AHA^21^ recommended cutoffs (ie, average office systolic BP ≥130 mm Hg or average office diastolic BP ≥80 mm Hg) for supplementary analyses. The ESH cutoffs for diagnosing hypertension are similar to those recommended by the European Society of Cardiology,^23^ the International Society of Hypertension,^24^ and the Pan African Society of Cardiology^10^ and are used in the diagnosis of hypertension for adults in SSA. Each guideline provides corresponding 24‐hour BP cutoffs, and these were used to determine participant hypertensive status by average 24‐hour BP from ABPM (ie, the reference standard). These BP cutoffs are summarized in Table S1.

Using the combination of uAOBP and average 24‐hour BP, we categorized participants into 4 hypertensive diagnostic phenotypes: sustained hypertension, masked hypertension, white‐coat hypertension, and sustained normotension. Participants with sustained hypertension were those who were classified as hypertensive on both uAOBP and average 24‐hour BP (true positives), those with white‐coat hypertension were classified as hypertensive on uAOBP but not on average 24‐hour BP (false positives), those with masked hypertension had normal uAOBP but were classified as hypertensive on average 24‐hour BP (false negatives), and those categorized as sustained normotension had normal BP on both uAOBP and average 24‐hour BP (true negatives).

In addition, we used ESH guidelines for hypertension on daytime and nighttime ABPM to determine the prevalence of hypertensive groups as follows: isolated nocturnal hypertension (nighttime BP of ≥120/70 mm Hg and a daytime BP <135/85 mm Hg), isolated daytime hypertension (daytime BP of ≥135/85 mm Hg and a nighttime BP of <120/70 mm Hg), sustained ambulatory hypertension (nighttime BP of ≥120/70 mm Hg and a daytime BP of ≥135/85 mm Hg), and sustained ambulatory normotension (nighttime BP <120/70 mm Hg and a daytime BP of <135/85 mm Hg).^25^

### Statistical Analysis

We determined the diagnostic performance of uAOBP for PWH and PWoH separately by comparing participant hypertensive status as determined by uAOBP against that determined by average 24‐hour BP from ABPM (reference standard). We calculated sensitivity, specificity, positive and negative predictive values, and positive and negative likelihood ratios for uAOBP with their corresponding 95% CI using ESH criteria for the primary analyses.^26^ Similar analyses using the ACC/AHA BP cutoffs were conducted as supplementary analyses. We used the area under the receiver‐operating characteristic curves (AUC) to determine overall diagnostic performance. Using the following point estimate criteria: ≥0.80, ≥0.60 to <0.80, and <0.60, we categorized the calculated measures of diagnostic performance as high, moderate, and low, respectively.

We used Bland–Altman plots to display the levels of agreement between uAOBP and (1) average 24‐hour BP (primary analyses) and (2) average daytime BP (supplementary analyses), as measured by ABPM, and the Pitman test to determine the correlation between the means and differences in these plots.^27^ We conducted stratified analyses of diagnostic performance by age and sex. We used a forward stepwise approach to build a multivariable logistic regression model to test the association between various predictors and a correct diagnosis of hypertension by uAOBP. The final model included HIV status, age group, sex, body mass index, and waist circumference. All analyses were conducted using STATA, version 16, software (Stata Corp., College Station, TX).

### Ethical Issues

This study was carried out in accordance with Good Clinical Practice and approved by the institutional review boards at both Weill Cornell Medicine and the Tanzanian National Health Research Ethics Committee. All participants provided written informed consent in Kiswahili. We offered lifestyle advice as recommended by the World Health Organization hearts package^28^ and referred participants identified as hypertensive to a local clinic and provided free access to medication.^29^

## RESULTS

### Characteristics of Included Participants

Between March 2022 and May 2023, 999 participants were recruited into the study, 959 (96%) of whom met criteria for a valid ABPM measurement (≥70% successful readings including ≥3 nighttime readings) and were included in the analyses. Of the 959, 483 (50.4%) were PWH and 476 (49.6%) were PWoH. Demographic characteristics were well balanced between participants by HIV status (Table 1). The median age was 44 years (interquartile range, 38–50 years) and 669 (69.8%) of the participants were women. Slightly more PWoH (193 [40.6%]) had overweight or obesity compared with PWH (161 [33.3%]) (Table 1).

**Table 1:** Distribution of Participants by Key Sociodemographic Parameters, Hypertensive Status, and Hypertensive Diagnostic Phenotypes

Participant groups		People with HIV	People without HIV
Sociodemographic characteristics		n=483, n (%)	n=476, n (%)
Sex	Female	340 (70.4)	329 (69.1)
Age	30–39 y	122 (25.3)	168 (35.3)
	40–49 y	218 (45.1)	186 (39.1)
	50–59 y	120 (24.8)	92 (19.3)
	>60 y	23 (4.8)	30 (6.3)
	Median age in y (IQR)	46 (39–50)	43 (36–50)
BMI	Underweight	47 (9.7)	39 (8.2)
	Healthy weight	275 (56.9)	244 (51.3)
	Overweight	102 (21.1)	127 (26.7)
	Obesity	59 (12.2)	66 (13.9)
	Median BMI (IQR)	22.5 (20.0–26.3)	23.5 (20.6–27.3)
Waist circumference	High†	156 (32.3)	152 (31.9)
Currently smoking	Yes	20 (4.1)	35 (7.4)
Currently taking alcohol	Yes	149 (30.8)	138 (29.0)
Ever diagnosed as high BP‡	Yes	66 (13.7)	64 (13.4)
On hypertensive medication	Yes	9 (1.9)	14 (2.9)
Unattended automated office BP (uAOBP)	Median systolic BP in mm Hg (IQR)	111 (102–122)	115 (104–127)
	Median diastolic BP in mm Hg (IQR)	68 (61–76)	71 (64–79)
Average 24‐hour BP	Median systolic BP in mm Hg (IQR)	76 (70–83)	77 (72–83)
	Median diastolic BP in mm Hg (IQR)	117 (111–125)	119 (112–127)
Hypertension diagnostic groups		% (95% CI)	% (95% CI)
People with hypertension	uAOBP	9.7 (7.2–12.7)	11.3 (8.6–14.5)
	Average 24‐hour BP	34.6 (30.3–39.0)	35.9 (31.6–40.4)
Hypertensive diagnostic phenotypes	Sustained normotension	64.6 (60.2–68.7)	62.0 (57.5–66.2)
	White‐coat hypertension	0.8 (0.3–2.2)	2.1 (1.1–3.9)
	Masked hypertension	25.7 (22.0–29.8)	26.7 (22.9–30.8)
	Sustained hypertension	8.9 (6.7–11.8)	9.2 (6.9–12.2)
Subgroups with hypertension based on average 24‐hour BP	Ambulatory normotension*	46.4 (42.0–50.9)	46.4 (42.0–50.9)
	Isolated daytime hypertension	4.8 (3.2–7.1)	4.0 (2.6–6.2)
	Isolated nocturnal hypertension	24.2 (20.6–28.3)	21.6 (18.2–25.6)
	Ambulatory hypertension*	24.6 (21.0–28.7)	27.9 (24.1–32.2)

* Ambulatory normotension refers to sustained daytime and nighttime normotension, and ambulatory hypertension refers to sustained daytime and nighttime hypertension on average 24‐hour BP.

^†^ Participant waist circumference was categorized as high if it was >88 cm in men and 100 cm in women.

^‡^ Participants were asked if they had ever been told by a doctor or other health care provider that they had high blood pressure.

BMI indicates body mass index; BP, blood pressure; IQR, interquartile range; and uAOBP, unattended automated office blood pressure.

### Hypertension and Hypertensive Diagnostic Phenotypes

Based on uAOBP at the ESH cutoffs, the prevalence of hypertension was 9.7% (95% CI, 7.2%–12.7%) for PWH and 11.3% (95% CI, 8.6%–14.5%) for PWoH. Based on average 24‐hour BP at the corresponding ESH cutoffs, the prevalence of hypertension was 34.6% (95% CI, 30.3%–39.0%) for PWH and 35.9% (95% CI, 31.6%–40.4%) for PWoH (Table 1).

**Figure 1:**
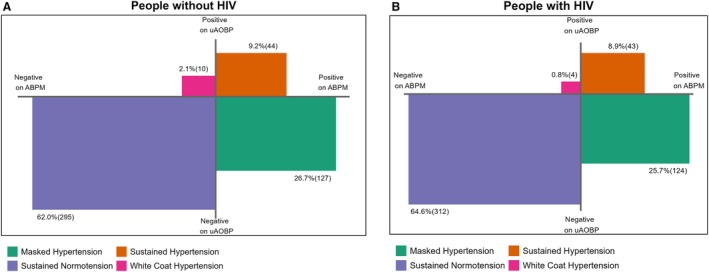
Hypertensive diagnostic phenotypes by HIV infection status at the ESH BP cutoffs for diagnosing hypertension. **A**, People without HIV; **B**, people with HIV. ABPM indicates ambulatory blood pressure monitoring; BP, blood pressure; ESH, European Society of Hypertension; and uAOBP, unattended automated office blood pressure.

The prevalence of masked hypertension was 25.7% (95% CI, 22.0%–29.8%) for PWH and 26.7% (95% CI, 22.9%–30.8%) for PWoH using ESH criteria. Prevalences of white‐coat hypertension were 0.8% (95% CI, 0.3%–2.2%) and 2.1% (95% CI, 1.1%–3.9%) for PWH and PWoH, respectively (Table 1, Figure 1, Table S3). There was no difference in the distribution of hypertensive diagnostic phenotypes by HIV status (χ^2^ *P*=0.385, Table S3). Based on average daytime and nighttime measurements, 24.2% (95% CI, 20.6%–28.3%) of PWH and 21.6% (95% CI, 18.2%–25.6%) of PWoH had isolated nocturnal hypertension. There was no difference in the distribution of groups with hypertension by HIV infection status (χ^2^ *P*=0.568).

### Diagnostic Performance of uAOBP

**Figure 2:**
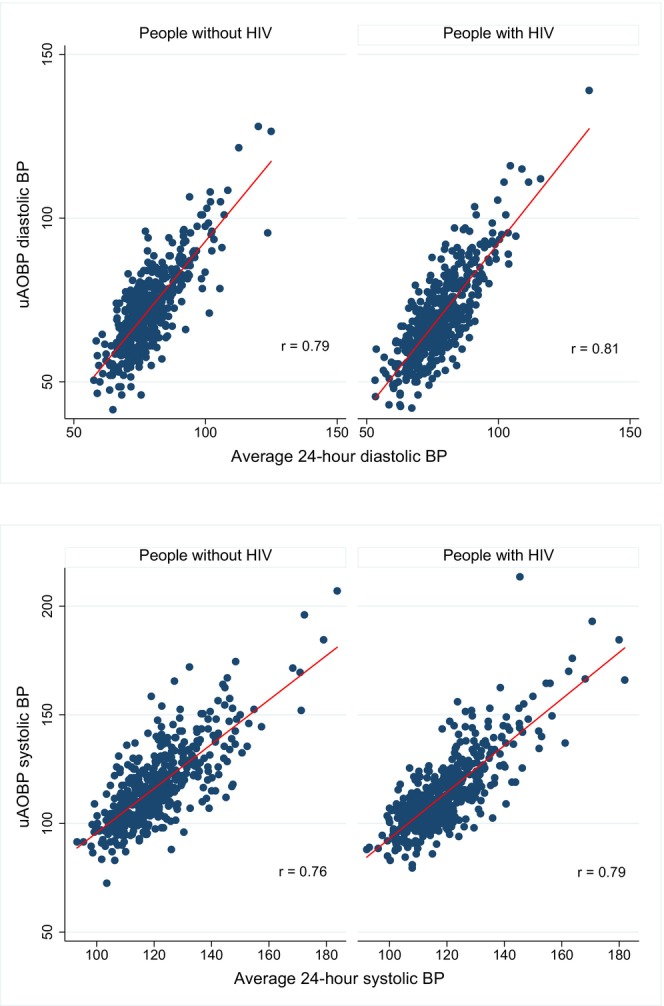
Correlation of systolic and diastolic uAOBP and average 24‐hour BP. The *r* in the scatter plots represents correlation coefficients for each plot. BP indicates blood pressure; and uAOBP, unattended automated office blood pressure.

**Figure 3:**
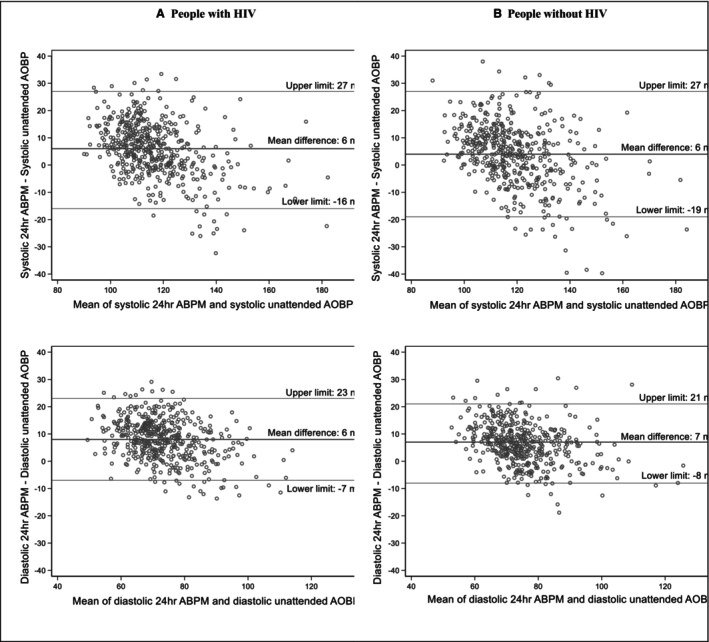
Bland–Altman plots of level of agreement for uAOBP and average 24‐hour BP for (A) people with HIV and (B) people without HIV. The correlation coefficients (Pitman test) between the difference and mean were *r*=−0.451 for systolic measurements and *r*=−0.363 for diastolic measurements for PWH and *r*=−0.420 for systolic measurements and *r*=−0.323 for diastolic measurements for PWoH. These suggest proportional bias for both systolic and diastolic measurements in both participant groups. Limits of agreement calculated as mean difference of uAOBP and average 24‐hour BP±1.96×SD of the differences. ABPM indicates ambulatory blood pressure monitoring; AOBP, automated office blood pressure; BP, blood pressure; PWH, people with HIV; and PWoH, people without HIV.

Measures of diagnostic performance did not differ significantly by HIV infection status, with uAOBP having a low sensitivity but high positive predictive value for each group (Table 2). The overall diagnostic performance of uAOBP as determined by the AUC was moderate at 0.62 (95% CI, 0.59–0.66) for PWH and 0.61 (95% CI, 0.58–0.65) for PWoH (Table 2). Although both systolic and diastolic uAOBP measurements were strongly correlated with corresponding average 24‐hour BP (Figure 2), Bland–Altman plots revealed poor agreement between average 24‐hour systolic and diastolic BP on ABPM and uAOBP measurements, with wide limits of agreement for both measurements for the 2 participant groups (Figure 3).

**Table 2:** Diagnostic Performance of uAOBP Among Participants by HIV Infection Status at the ESH Cutoffs for Diagnosing Hypertension

Diagnostic performance measure	Participant groups by HIV infection status % (95% CI)	
	People with HIV	People without HIV
Sensitivity	25.7 (19.3–33.1)	25.7 (19.4–33.0)
Specificity	98.7 (96.8–99.7)	96.7 (94.1–98.4)
Positive predictive value	91.5 (79.6–97.6)	81.5 (68.6–90.7)
Negative predictive value	71.6 (67.1–75.8)	69.9 (65.3–74.2)
Area under the receiver‐operating characteristic curve	0.62 (0.59–0.66)	0.61 (0.58–0.65)
Likelihood ratio positive	20.3 (7.43–55.7)	7.85 (4.05,15.2)
Likelihood ratio negative	0.75 (0.69–0.82)	0.77 (0.71–0.84)
Diagnostic odds ratio	27.0 (9.9–73.8)	10.2 (5.1–20.7)

This table summarizes the measures of diagnostic performance for uAOBP when compared with average 24‐hour blood pressure. ESH indicates European Society of Hypertension; and uAOBP, unattended automated office blood pressure.

### Stratified Analysis and Predictors of Diagnostic Performance

There was no difference in overall diagnostic performance for uAOBP as measured by AUC among the 2 participant groups when stratified by age group and sex at the ESH BP cutoffs for diagnosing hypertension (Tables S4–S6). Based on multivariable logistic regression analysis: older age (*P*<0.001), male sex (*P*<0.001), and higher body mass index (*P*=0.002) increased the likelihood of being correctly identified as hypertensive by uAOBP (Table S7).

### Supplementary Analyses

When ACC/AHA BP cutoffs for diagnosing hypertension were considered, prevalences of hypertension on uAOBP were 20.1% (95% CI, 16.6%–23.9%) for PWH and 26.7% (95% CI, 22.8%–30.9%) for PWoH, and those from average 24‐hour BP were 55.7% (95% CI, 51.1%–60.2%) and 62.2% (95% CI, 57.7%–66.6%), respectively (Table S8). Prevalences of masked hypertension at the ACC/AHA BP cutoffs were higher than those at the ESH BP cutoffs, at 36.0% (95% CI, 31.9%–40.4%) for PWH and 37.0% (95% CI, 32.7%–41.4%) for PWoH (Table S9), and the proportion of participants having isolated nocturnal hypertension remained high at 29.4% (95% CI, 25.5%–33.6%) for PWH and 27.3% (95% CI, 23.5%– 31.5%) for PWoH (Table S10). Overall diagnostic performance of uAOBP compared with average 24‐hour BP at the ACC/AHA BP cutoffs was similar to that of the ESH BP cutoffs (Table S11).

Supplementary analyses of hypertensive diagnostic phenotypes, diagnostic performance, and levels of agreement of uAOBP using average daytime BP as the reference standard revealed similar results with low to moderate diagnostic performance and wide limits of agreement on Bland–Altman plots (Tables S12 and S13, Figure S1).

## DISCUSSION

We report a high prevalence of hypertension (35%–36%) in a relatively young population in Tanzania as measured by ABPM with no differences in prevalences by HIV infection status. Second, we report a wide and statistically significant difference in the prevalence of hypertension as determined by uAOBP measurements compared with that determined by average 24‐hour BP on ABPM. This difference partially explains the low sensitivity and moderate overall discrimination (as measured by AUC) of uAOBP. Masked hypertension was found in more than one quarter of participants, and isolated nocturnal hypertension was present in >20%. We did not observe statistically significant differences in measures of diagnostic performance by HIV infection status at different BP cutoffs, an important finding as there are no specific guidelines for the measurement of BP among PWH. Although uAOBP's specificity was high, contributing to a moderate AUC, more than two‐thirds of all participants with true hypertension, as diagnosed by average 24‐hour BP on ABPM, were missed by uAOBP at the ESH or ACC/AHA BP cutoffs for diagnosing hypertension.

As the global burden of hypertension increases, regular screening for elevated BP with attended or unattended AOBP measurements is encouraged by the World Health Organization and international guidelines.^3^ The use of uAOBP in clinical research has gained much popularity after the SPRINT (Systolic Blood Pressure Intervention Trial) trial.^30^ However, its value in clinical practice is still unclear as there are limited data.^31^ These questions are particularly relevant in SSA where there are limited data on the diagnostic performance of BP measurement methods and whose population is younger, has a lower body mass index, and has been reported to experience more nocturnal hypertension^11^ compared with the populations informing current hypertension guidelines.

Our results of uAOBP's overall diagnostic performance are similar to those reported by the only other study reporting similar analyses in SSA.^32^ In this study, Etyang et al. reported a slightly higher sensitivity (44%) and lower positive predictive value (68%) but a similar AUC (0.66) for uAOBP at the ESH BP cutoffs for diagnosing hypertension.^32^ Our results are strikingly similar to another study comparing uAOBP and ABPM in South Korea.^33^ According to Bland–Altman analysis, agreement was poor in both studies, although the limits of agreement were slightly narrower in our study. Both studies showed slight overall bias toward lower uAOBP versus ABPM (6–7 mm Hg), but this bias appeared to differ by average BP with a lower uAOBP versus ABPM at lower average BP and vice‐versa.

A notable finding in our study was the high prevalence of masked hypertension. More than 25% of all participants had masked hypertension when comparing uAOBP with 24‐hour ABPM. Our reported prevalence of masked hypertension would have been even higher if we used all 3 sets of criteria for hypertension by ABPM (24 hour, awake, and asleep). Masked hypertension has received increased attention in recent years with many studies noting prevalence by ethnicity and the presence of comorbidities like HIV and renal disease.^15^, ^34^, ^35^, ^36^ Among adults without HIV in SSA, the prevalence of masked hypertension ranges between 11%^37^ and 18%^34^ whereas recent estimates among PWH report the prevalence being about 32%.^38^ Ours is the largest comparative study reporting masked hypertension prevalence in PWH compared with PWoH. Our results indicate that masked hypertension is common in both PWH and PWoH in SSA, which is concerning as the diagnosis of masked hypertension in SSA is limited by access to and availability of ABPM.^10^, ^34^

Masked hypertension is particularly important as it has been shown to carry a similar cardiovascular risk to sustained hypertension but remains underdiagnosed leading to undertreatment and poor cardiovascular outcomes.^34^, ^37^ To increase screening and diagnosis of masked hypertension, evaluating low‐cost strategies for the detection of masked hypertension such as self‐applied nocturnal BP measurements^39^ or use of wrist‐based devices^40^ within the SSA setting is crucial. Such interventions will be particularly useful in settings where ABPM is not accessible. In addition, longitudinal studies are needed to determine the long‐term cardiovascular risk for patients who have masked hypertension in SSA.

In our study population, uAOBP does indeed appear to be useful in reducing white‐coat hypertension, but this method still falls short in identifying all hypertension diagnoses identified by ABPM. On the one hand, these results are encouraging for resource‐limited settings where it is critical to initiate antihypertensive medications only on patients who truly have hypertension and will benefit from treatment. On the other hand, these results indicate the need to increase the access to and availability of out‐of‐office measurements in SSA for people with normal office BP but high CVD risk.^37^

### Strengths and Limitations

Given the limited data on the diagnostic performance of BP measurements methods in SSA, our study's main strength is that it provides these data for a high‐risk population with CVD, PWH, and compares this performance to PWoH. We included a large sample size with over 900 valid ABPM measurements and comparative uAOBP measurements that improve our understanding of the relationship between clinic uAOBP and 24‐hour ABPM among populations in SSA. Second, most studies evaluating diagnostic performance have limited their analyses to one of the major guidelines on screening and diagnosis of hypertension. We compared uAOBP with average 24‐hour BP at 2 major guidelines on screening and diagnosis of hypertension, the ESH and ACC/AHA guidelines.

The findings of this study are cross‐sectional. Analyses from single uAOBP measurements may not be enough to conclusively determine uAOBP's value in clinical practice in SSA and other low‐ and middle‐income countries. As there are no data from SSA on the strength of association between office and out‐of‐office BP measurements and clinical outcomes in SSA, comparative longitudinal analysis of the short‐term and long‐term reproducibility of uAOBP and its relationship with incident cardiovascular morbidity and mortality is urgently needed.

## CONCLUSIONS

We report a moderate diagnostic performance of uAOBP, with no differences by HIV infection status. Masked hypertension, and particularly isolated nocturnal hypertension, were common, but white‐coat hypertension was rare. Our results suggest that uAOBP might be a reasonable screening tool in resource‐limited settings to identify those patients who almost certainly will benefit from hypertension treatment. On the other hand, low‐cost strategies for out‐of‐office BP measurement are needed to detect masked hypertension, particularly for people with high CVD risk such as PWH, because they would also likely benefit from hypertension treatment. Given the limited data on the diagnostic performance of AOBP in SSA, future studies should answer questions on the short‐ and long‐term reproducibility of AOBP measurements (both attended and unattended) and their association with clinical outcomes.

## Sources of Funding

This study is funded by a grant from the National Heart, Lung, and Blood Institute (R01 HL160332). In addition, Robert N. Peck is supported by the National Heart, Lung, and Blood Institute (K24 HL170902). Benson Issarow, Salama Fadhil, Godfrey Kisigo, and Saidi Kapiga are supported by the Fogarty International Center (D43 TW011826). Anthony O. Etyang and Ruth K. Lucinde are supported by the National Institute of Health and Care Research (NIHR 134544) and Wellcome 22 713/Z/23/Z. This research was funded in part by Science for Africa Foundation to the Developing Excellence in Leadership, Training, and Science in Africa (DELTAS Africa) program (DEL‐22‐012) with support from Wellcome Trust and the UK Foreign, Commonwealth & Development Office and is part of the EDCPT2 program supported by the European Union. For purposes of open access, the author has applied a CC BY public copyright license to any Author Accepted Manuscript version arising from this submission. The content is solely the responsibility of the authors and does not necessarily represent the official views of the National Institutes of Health or other funding agencies.

## Disclosures

None.

## Supporting information

Data S1
Tables S1–S13
Figures S1–S3
