# Supplementary material for: Diagnostic Performance of Unattended Automated Office Blood Pressure Measurement for Hypertension Screening Among People With and Without HIV
Source: J Am Heart Assoc. 2025 Sep 19;14(19):e043957. doi: 10.1161/JAHA.125.043957 (PMC7618255; doi:10.1161/JAHA.125.043957)
Supplement: Supplementary file 1 — Data S1 Tables S1–S13 Figures S1–S3 [file JAH3-14-e043957-s001.pdf]

Supplemental Material

Table S1: Recommended blood pressure cutoffs for diagnosing hypertension based on AOBP and 24-hour ABPM. This table summarizes the BP cutoffs for automated office blood pressure (AOBP) for diagnosing hypertension recommended by the European Society of Hypertension (ESH) and the joint American College of Cardiology/American Heart Association. For each guideline's recommended AOBP's BP cutoff, a corresponding ABPM cutoff is provided.

**Guidelines**	**AOBP cutoff**	**Average 24-hour BP cutoff**	**Average awake BP cutoff**	**Average asleep BP cutoff**
ESH guidelines	140/90 mmHg	130/80 mmHg	135/85 mmHg	120/70 mmHg
ACC/AHA guidelines	130/80 mmHg	125/75 mmHg	130/80 mmHg	110/65 mmHg
*Average 24-hour BP was used as the reference standard measurement for analyses in this manuscript				

Table S2: Prevalence of hypertension based on 24- hour ABPM parameters at the ESH BP cutoffs for diagnosing hypertension. The table below summarizes the proportion of participants meeting the criteria for hypertension based on average daytime BP, average nighttime, average 24-hour BP and overall ABPM at the ESH cutoffs for diagnosing hypertension on ABPM

**24-hour ABPM parameters**	**People with HIV n (%)**	**People without HIV n (%)**
Average daytime BP	142 (29.4)	152 (31.9)
Average nighttime BP	236 (48.9)	236 (49.6)
Average 24-hour BP	167 (34.6)	171 (35.9)
Overall ABPM	260 (53.8)	256 (53.8)

Table S3: Hypertensive diagnostic phenotypes for uAOBP based on the ESH cutoffs for diagnosing hypertension. The table below summarizes the hypertensive diagnostic phenotypes when uAOBP is compared to average 24-hour BP at the ESH BP cutoffs for diagnosing hypertension. P values were generated from a chi square test comparing the distribution of participants' hypertensive diagnostic phenotypes by HIV infection status.

**Hypertension diagnostic phenotype**	**People with HIV n (%)**	**People without HIV n (%)**	**Chi square p value**
Sustained normotension	312 (64.6)	295 (62.0)	0.385
White-coat hypertension	4 (0.8)	10 (2.1)	
Masked hypertension	124 (25.7)	127 (26.7)	
Sustained hypertension	43 (8.9)	44(9.2)	

Table S4: Hypertensive diagnostic phenotypes for uAOBP and hypertensive subgroups based on the ESH cutoffs for diagnosing hypertension. The table below summarizes the overall distribution of hypertensive subgroups (based on average asleep and awake readings on ABPM) for the four hypertensive diagnostic phenotypes when uAOBP is compared to average 24-hour BP at the ESH BP cutoffs for diagnosing hypertension.

**Hypertensive diagnostic phenotypes**	**Hypertensive subgroups**			
	Isolated nocturnal hypertension n (%)	Isolated daytime hypertension n (%)	Ambulatory hypertension n (%)	Ambulatory normotension n (%)
Sustained normotension	155 (25.5)	11 (1.8)	0 (0.0)	441 (72.7)
Whitecoat hypertension	9 (64.3)	2 (14.3)	1 (7.1)	2 (14.23)
Masked hypertension	55 (21.9)	27 (10.8)	167 (66.5)	2 (0.8)
Sustained hypertension	1 (1.2)	2 (2.3)	84 (96.6)	0 (0.0)

<u>Stratified analyses of diagnostic performance at the ESH BP cutoffs</u>

Table S5: Diagnostic performance of uAOBP compared to average 24-hour BP stratified by age and HIV infection status

Age category	Prevalence % (95% CI)	Sensitivity % (95% CI)	Specificity % (95% CI)	PPV % (95% CI)	NPV % (95% CI)	AUC % (95% CI)
**People without HIV**						
30 – 39 years	22.0 (16.0, 29.1)	40.5 (24.8, 57.9)	92.4 (86.4, 96.3)	60.0 (38.7, 78.9)	84.6 (77.6, 90.1)	0.67 (0.58, 0.75)
40 – 49 years	36.0 (29.0, 43.4)	59.7 (47.0, 71.5)	95.0 (89.3, 98.1)	87.0 (73.7, 95.1)	80.7 (73.2, 86.9)	0.77 (0.71, 0.84)
50 – 59 years	55.0 (45.0, 65.8)	58.8 (44.2, 72.4)	78.0 (62.4, 89.4)	76.9 (60.7, 88.9)	60.4 (46.0, 73.5)	0.68 (0.59, 0.78)
More than 60 years	53.0 (34.0, 71.7)	81.3 (54.4, 96.0)	71.4 (41.9, 91.6)	76.5 (50.1, 93.2)	76.9 (46.2, 95.0)	0.76 (0.61, 0.92)
**People with HIV**						
30 – 39 years	23.0 (16.0, 31.4)	42.9 (24.5, 62.8)	97.9 (92.5, 99.7)	85.7 (57.2, 98.2)	85.2 (77.1, 91.3)	0.71 (0.61, 0.80)
40 – 49 years	31.0 (25.0, 37.3)	53.7 (41.1, 66.0)	96.0 (91.6, 98.5)	85.7 (71.5, 94.6)	82.4 (75.9, 87.7)	0.75 (0.69, 0.81)
50 – 59 years	48.0 (39.0, 57.6)	50.0 (36.6, 63.4)	95.2 (86.5, 99.0)	90.6 (75.0, 98.0)	67.0 (56.2, 76.7)	0.73 (0.66, 0.80)
More than 60 years	61.0 (39.0, 80.3)	57.1 (28.9, 82.3)	88.9 (51.8, 99.7)	88.9 (51.8, 99.7)	57.1 (28.9, 82.3)	0.73 (0.56, 0.91)

Table S6: Diagnostic performance of uAOBP compared to average 24-hour BP stratified by sex and HIV infection status. The table below summarizes the measures of diagnostic performance for uAOBP stratified by sex for people with and without HIV when compared to average 24-hour BP.

Diagnostic performance measure	People with HIV		People without HIV	
	Male n (95% CI)	Female n (95% CI)	Male n (95% CI)	Female n (95% CI)
**Prevalence**	47.0 (38.0, 55.4)	29.0 (25.0, 34.6)	46.0 (28.0, 54.7)	31.0 (26.0, 36.6)
**Sensitivity**	100 (95.3,100)	31.0 (22.1, 41.0)	13.2 (6.23, 23.6)	34.0 (24.9, 44.0)
**Specificity**	17.9 (9.61, 29.2)	98.3 (95.8, 99.5)	94.9 (87.5, 98.6)	97.3 (94.3, 99.0)
**Positive predictive value**	100 (73.5,100)	88.6 (73.3, 96.8)	69.2 (38.6, 90.9)	85.4 (70.8, 94.4)
**Negative predictive value**	58.0 (49.1, 66.6)	77.4 (72.3, 82.0)	56.0 (47.1, 64.5)	76.4 (71.1, 81.2)
**AUC**	0.59 (0.54, 0.64)	0.65 (0.60, 0.69)	0.54 (0.49, 0.59)	0.66 (0.61, 0.70)
**Likelihood Ratio positive**	-	18.6 (6.74, 51.3)	2.61 (0.84, 8.11)	12.8 (5.56, 29.5)
**Likelihood Ratio negative**	0.82 (0.73,0.92)	0.70 (0.62, 0.80)	56.0 (47.1, 64.5)	0.68 (0.59, 0.78)

AUC – area under receiver operating characteristic curve

'-' Likelihood ratio positive cannot be calculated as the positive predictive value and specificity are 100%

Multivariable logistic regression analysis of predictors of hypertension diagnosis

Table S7: Predictors of hypertension diagnosis by uAOBP. The table below summarizes the results of a multivariable logistic regression model evaluating the predictors of a higher likelihood of having a diagnosis of hypertension when BP is measured by uAOBP.

Sociodemographic characteristics	Odds Ratio (OR)	95% confidence interval	p-value
**HIV infection status**			
People with HIV	-		
People without HIV	1.04	0.78, 1.38	0.810
**Age Group**			
30-39 years	-		
40-49 years	1.40	1.01, 1.96	0.045
50-59 years	2.56	1.70, 3.86	<0.001
More than 60 years	4.72	2.10, 10.64	<0.001
**Sex**			
Female	-		
Male	2.72	1.94, 3.81	<0.001
**Body mass index (BMI)**			
Underweight	-		
Healthy weight	1.69	1.00, 2.84	0.049
Overweight	3.00	1.56, 5.75	0.001
Obesity	3.06	1.42, 6.59	0.004

Supplementary analyses of diagnostic performance using the ACC/AHA guidelines for diagnosing hypertension

Table S8: Prevalence of hypertension based on 24- hour ABPM parameters at the ACC/AHA BP cutoffs for diagnosing hypertension

24-hour ABPM parameters	People without HIV n (%)	People with HIV n (%)
Average daytime BP	244 (51.3)	227 (47.0)
Average nighttime BP	360 (75.6)	355 (73.5)
Average 24-hour BP	296 (62.2)	269 (55.7)
Overall ABPM	375 (78.8)	370 (76.6)

Table S9: Hypertensive diagnostic phenotypes for uAOBP based on the ACC/AHA cutoffs for diagnosing hypertension. The table below summarizes the hypertensive diagnostic phenotypes when uAOBP is compared to average 24-hour BP at the ACC/AHA recommended BP cutoffs. P values were generated from a chi square test comparing the distribution of participants' hypertensive diagnostic phenotypes by HIV infection status.

**Hypertension diagnostic phenotype**	**People with HIV n (%)**	**People without HIV n (%)**	**Chi square p value**
Normotensive	173 (36.3)	212 (43.9)	0.022
White Coat Hypertension	7 (1.5)	2 (0.4)	
Masked hypertension	176 (37.0)	174 (36.0)	
Sustained hypertension	120 (25.2)	95 (19.7)	

Table S10: Hypertensive groups based on average 24-hour BP at the ACC/AHA cutoffs for diagnosing hypertension. The table below summarizes the hypertensive groups based on when BP is above ACC/AHA cutoffs for diagnosing hypertension on average 24-hour BP. P values were generated from a chi square test comparing the distribution of participants' hypertensive groups by HIV infection status.

Hypertension diagnostic phenotype	People with HIV n (%)	People without HIV n (%)	Chi square p value
* Ambulatory normotension	114 (23.6)	102 (21.4)	0.615
Isolated daytime hypertension	14 (2.9)	14 (2.9)	
Isolated nocturnal hypertension	142 (29.4)	130 (27.3)	
* Ambulatory hypertension	213 (44.1)	230 (48.3)	

* Ambulatory normotension refers to sustained daytime and nighttime normotension while ambulatory hypertension refers to sustained daytime and nighttime hypertension on average 24-hour BP

Table S11: Diagnostic performance of uAOBP among included participants by HIV infection status at the ACC/AHA cutoffs for diagnosing hypertension. This table summarizes the measures of diagnostic performance for uAOBP when compared to average 24-hour BP on ABPM

**Diagnostic performance measure**	**Participant groups by HIV infection status n (95% CI)**	
	**People with HIV**	**People without HIV**
**Prevalence**	56.0 (51.0, 60.2)	62.0 (58.0, 66.6)
**Sensitivity**	35.3 (29.6, 41.4)	40.5 (34.9, 46.4)
**Specificity**	99.1 (96.7, 99.9)	96.1 (92.2, 98.4)
**Positive predictive value**	97.9 (92.7, 99.7)	94.5 (89.0, 97.8)
**Negative predictive value**	54.9 (49.8, 60.0)	49.6 (44.2, 54.9)
**AUC**	0.67 (0.64, 0.70)	0.68 (0.65, 0.72)
**Likelihood Ratio positive**	37.8 (9.42, 152)	10.4 (4.98, 21.8)
**Likelihood Ratio negative**	0.65 (0.60, 0.71)	0.62 (0.56, 0.68)

AUC – area under receiver operating characteristic curve

<u>Supplementary analysis of diagnostic performance of uAOBP average daytime BP as the reference standard</u>

Table S12: Hypertensive diagnostic phenotypes for uAOBP when compared to average daytime BP at the ESH cutoffs for diagnosing hypertension.

**Hypertension diagnostic phenotype**	**People with HIV n (%)**	**People without HIV n (%)**	**Chi square p value***
Normotensive	337 (69.8)	316 (66.4)	0.513
White Coat Hypertension	4 (0.8)	8 (1.2)	
Masked hypertension	99 (20.5)	106 (22.3)	
Sustained hypertension	43 (8.9)	46 (9.7)	

* P values were generated from a chi square test comparing the distribution of participants' hypertensive diagnostic phenotypes by HIV infection status

Table S13: Diagnostic performance of uAOBP among included participants by HIV infection status using average daytime BP as the reference standard at the ESH cutoffs for diagnosing hypertension.

Diagnostic performance measure	Participant groups by HIV infection status n (95% CI)	
	People with HIV	People without HIV
Prevalence	29.0 (25.0, 33.7)	32.0 (28.0, 36.3)
Sensitivity	30.3 (22.9, 38.5)	30.3 (23.1, 38.2)
Specificity	98.8 (97.0, 99.7)	97.5 (95.2, 98.9)
Positive predictive value	91.5 (79.6, 97.6)	85.2 (72.9, 93.4)
Negative predictive value	77.3 (73.1, 81.1)	74.9 (70.5, 79.0)
AUC	0.65 (0.61, 0.68)	0.64 (0.60, 0.68)
Likelihood Ratio positive	25.8 (9.44, 70.6)	12.3 (5.93, 25.3)
Likelihood Ratio negative	0.71 (0.63, 0.79)	0.72 (0.64, 0.80)

AUC – area under receiver operating characteristic curve

Figure S1: Correlation of A. systolic and B. diastolic uAOBP measurements and average daytime BP on ABPM

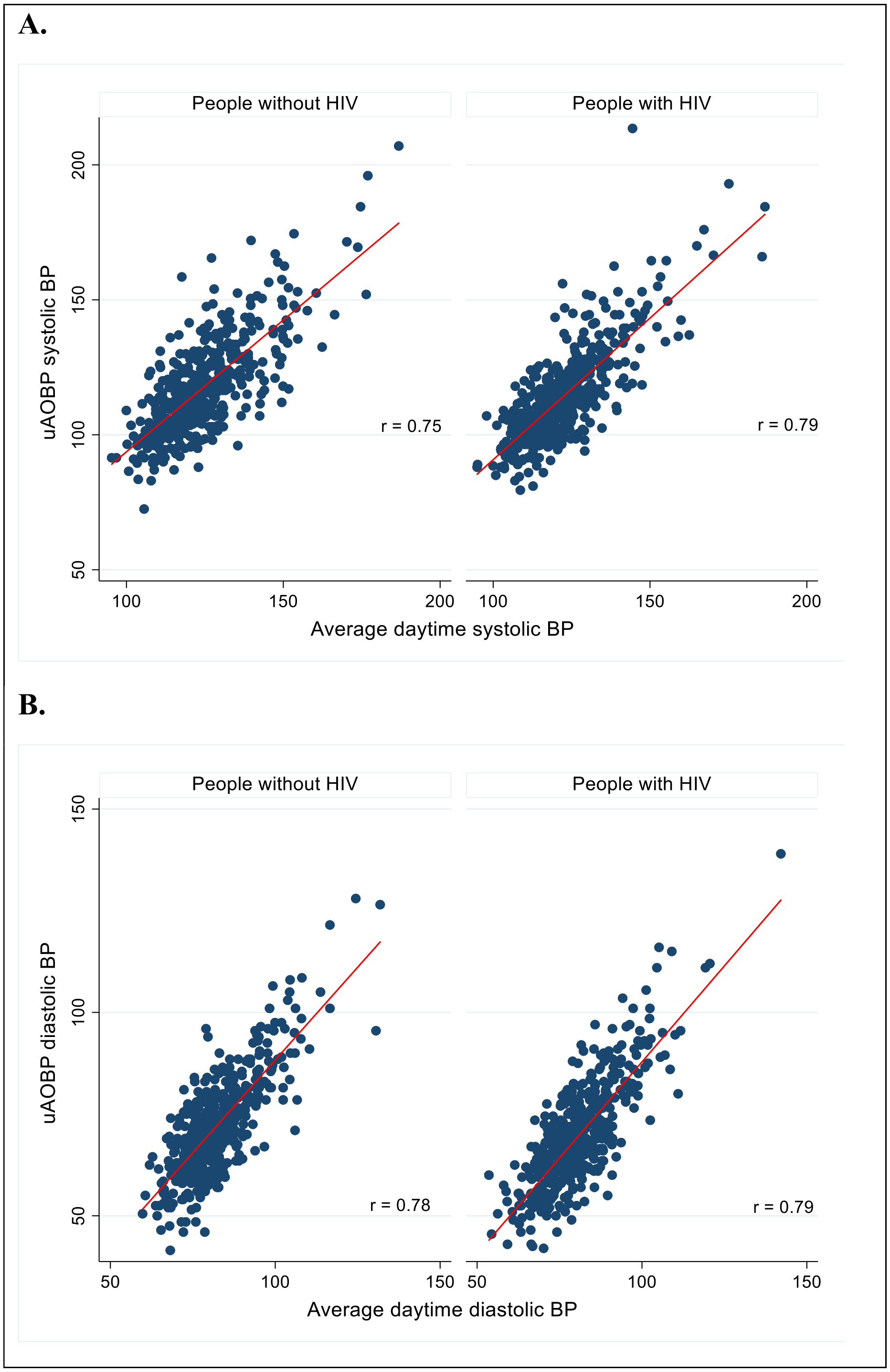

Figure S2: Bland Altman plots of level of agreement for A. systolic and B. diastolic uAOBP and average daytime BP on ABPM for people without HIV.

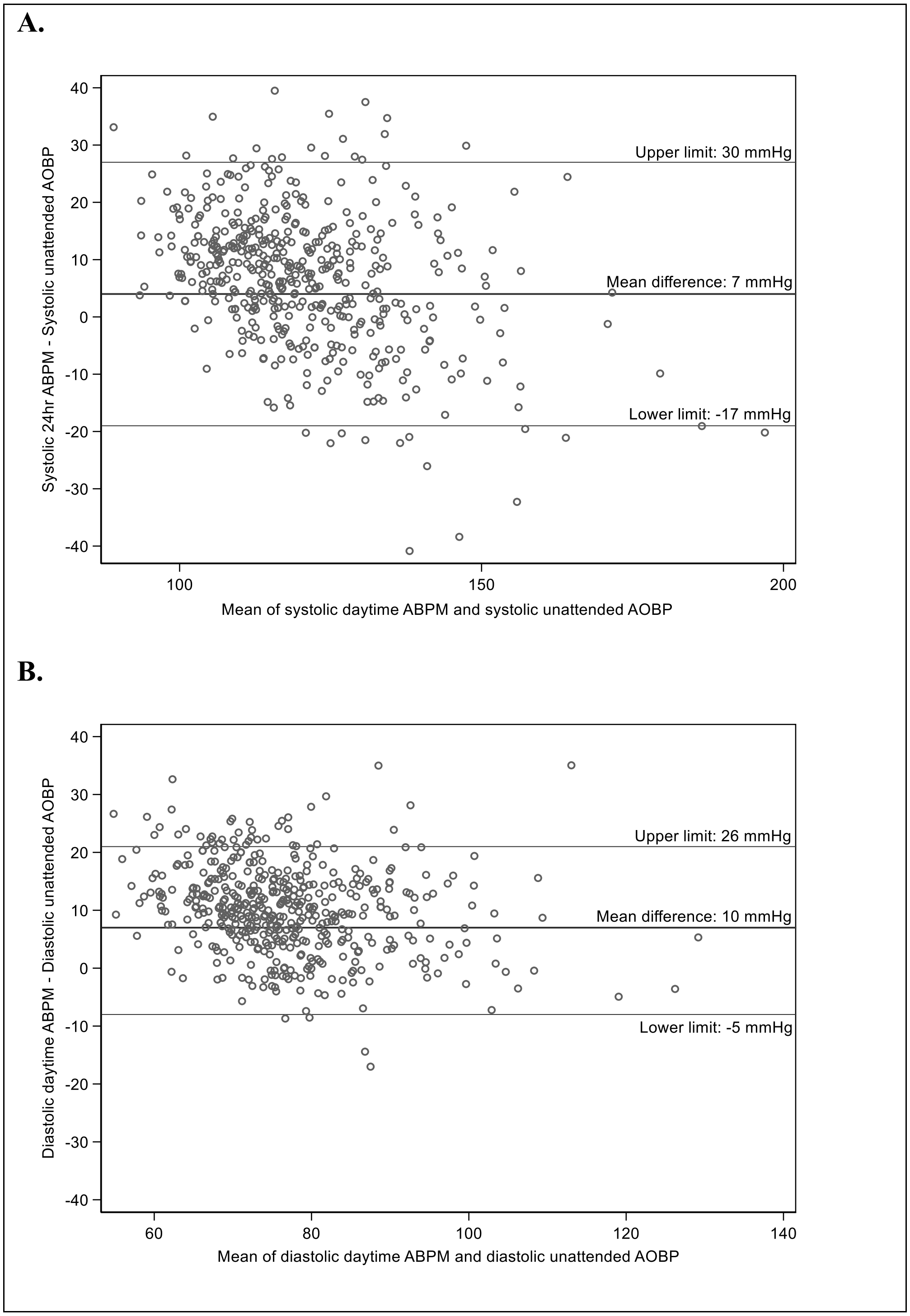

The correlation coefficients (Pitman test) between the difference and mean were r=−0.372, P= <0.001 for systolic measurements and r=-0.247, P= <0.001 for diastolic measurements.

Figure S3: Bland Altman plots of level of agreement for A. systolic and B. diastolic uAOBP and average daytime BP on ABPM for people with HIV.

A.

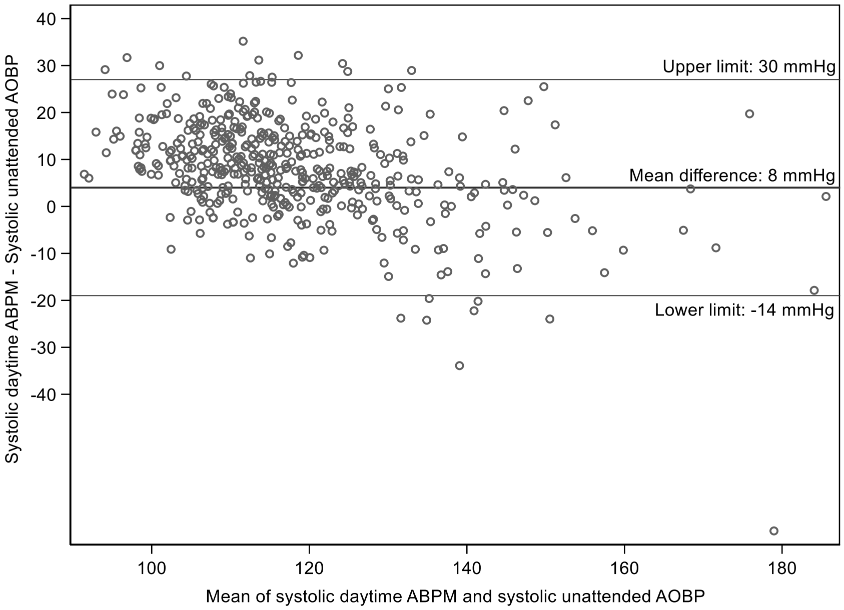

B.

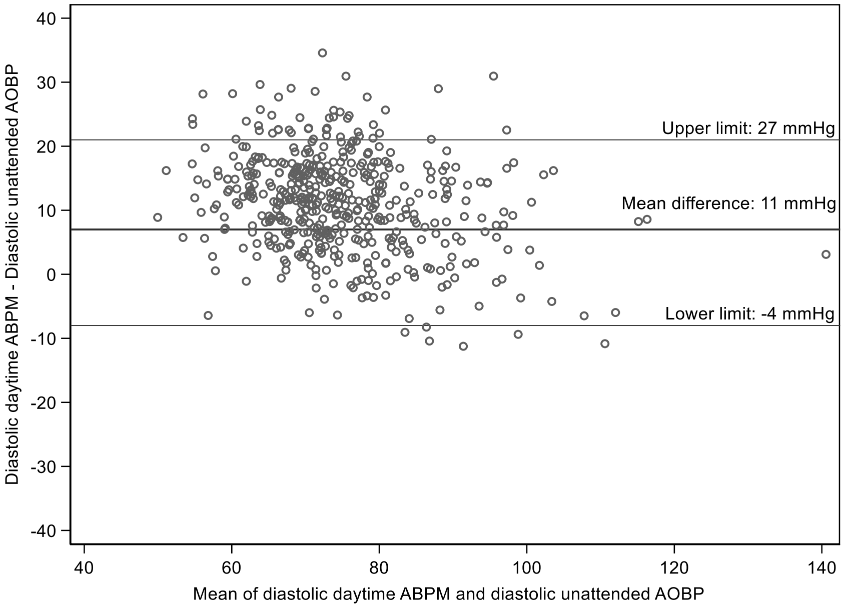

The correlation coefficients (Pitman test) between the difference and mean were r=−0.428, P= <0.001 for systolic measurements and r=-0.277, P= <0.001 for diastolic measurements.
