# Supplementary material for: Diagnostic Performance of Unattended Automated Office Blood Pressure Measurement for Hypertension Screening Among People With and Without HIV
Source: J Am Heart Assoc. Author manuscript; Available in PMC 2025 Oct 15. (PMC7618255; doi:10.1161/JAHA.125.043957)
Supplement: Supplementary file 1 [file EMS209559-supplement-Supplement.docx]

**Supplemental Material**

**Diagnostic performance of unattended automated office blood pressure measurement for hypertension screening among people with and without HIV**

**Authors:**

Ruth K Lucinde MD MSc^1^, Megan Willkens BS^2^, Benson Issarow MD MSc^3^, Salama Fadhil BS^3^, Cody Cichowitz MD MPH^4^, Philip Ayieko PhD^3^, Godfrey Kisigo MD MSc^3^,^5^, Sara Venkatraman PhD MS^2^, Heiner Grosskurth MD PhD^3^,^5^, Ana Krieger MD^6^, Richard Devereux MD^7^, Myung Hee Lee PhD^2^, Saidi Kapiga MD ScD^3^,^5^ *Robert Peck MD PhD^2,3,8^, *Anthony O Etyang MD PhD^1^

*equal contribution

**Affiliations:**

^1 ^Department of Epidemiology and Demography, KEMRI-Wellcome Trust Research Programme, Kilifi, Kenya

^2^ Center for Global Health, Department of Medicine, Weill Cornell Medicine, New York, New York, USA 

^3^ Mwanza Intervention Trials Unit, National Institute of Medical Research, Mwanza, Tanzania

^4^ Division of Cardiology, Department of Medicine, University of California San Francisco, San Francisco, California, USA

^5^ Department of Infectious Disease Epidemiology, London School of Hygiene and Tropical Medicine, London, UK

^6^ Weill Cornell Center for Sleep Medicine, Department of Medicine, Weill Cornell Medical College, New York, New York, United States

^7^ Division of Cardiology, Department of Medicine, Weill Cornell Medicine, New York, New York, USA

^8^ Weill Bugando School of Medicine, Department of Medicine, Catholic University of Health and Allied Sciences, Mwanza, Tanzania

**Table of Contents**

Table S1: Recommended blood pressure cutoffs for diagnosing hypertension based on AOBP and 24-hour ABPM.	3

Table S2: Prevalence of hypertension based on 24- hour ABPM parameters at the ESH BP cutoffs for diagnosing hypertension.	3

Table S3: Hypertensive diagnostic phenotypes for uAOBP based on the ESH cutoffs for diagnosing hypertension.	4

Table S4: Hypertensive diagnostic phenotypes for uAOBP and hypertensive subgroups based on the ESH cutoffs for diagnosing hypertension.	4

Stratified analyses of diagnostic performance at the ESH BP cutoffs	5

Table S5: Diagnostic performance of uAOBP compared to average 24-hour BP stratified by age and HIV infection status	5

Table S6: Diagnostic performance of uAOBP compared to average 24-hour BP stratified by sex and HIV infection status.	6

Multivariable logistic regression analysis of predictors of hypertension diagnosis	7

Table S7: Predictors of hypertension diagnosis by uAOBP.	7

Supplementary analyses of diagnostic performance using the ACC/AHA guidelines for diagnosing hypertension	8

Table S8: Prevalence of hypertension based on 24- hour ABPM parameters at the ACC/AHA BP cutoffs for diagnosing hypertension	8

Table S9: Hypertensive diagnostic phenotypes for uAOBP based on the ACC/AHA cutoffs for diagnosing hypertension.	8

Table S10: Hypertensive groups based on average 24-hour BP at the ACC/AHA cutoffs for diagnosing hypertension.	9

Table S11: Diagnostic performance of uAOBP among included participants by HIV infection status at the ACC/AHA cutoffs for diagnosing hypertension.	10

Supplementary analysis of diagnostic performance of uAOBP average daytime BP as the reference standard	11

Table S12: Hypertensive diagnostic phenotypes for uAOBP when compared to average daytime BP at the ESH cutoffs for diagnosing hypertension.	11

Table S13: Diagnostic performance of uAOBP among included participants by HIV infection status using average daytime BP as the reference standard at the ESH cutoffs for diagnosing hypertension.	12

Figure S1: Correlation of A. systolic and B. diastolic uAOBP measurements and average daytime BP on ABPM	13

Figure S2: Bland Altman plots of level of agreement for A. systolic and B. diastolic uAOBP and average daytime BP on ABPM for people without HIV.	14

Figure S3: Bland Altman plots of level of agreement for A. systolic and B. diastolic uAOBP and average daytime BP on ABPM for people with HIV.	15

Table S1: Recommended blood pressure cutoffs for diagnosing hypertension based on AOBP and 24-hour ABPM. This table summarizes the BP cutoffs for automated office blood pressure (AOBP) for diagnosing hypertension recommended by the European Society of Hypertension (ESH) and the joint American College of Cardiology/American Heart Association. For each guideline’s recommended AOBP’s BP cutoff, a corresponding ABPM cutoff is provided. 

| **Guidelines** | **AOBP cutoff** | **Average 24-hour BP cutoff** | **Average awake BP cutoff** | **Average asleep BP cutoff** |
| --- | --- | --- | --- | --- |
| ESH guidelines | 140/90 mmHg | 130/80 mmHg | 135/85 mmHg | 120/70 mmHg |
| ACC/AHA guidelines | 130/80 mmHg | 125/75 mmHg | 130/80 mmHg | 110/65 mmHg |
| *Average 24-hour BP was used as the reference standard measurement for analyses in this manuscript |  |  |  |  |

Table S2: Prevalence of hypertension based on 24- hour ABPM parameters at the ESH BP cutoffs for diagnosing hypertension. The table below summarizes the proportion of participants meeting the criteria for hypertension based on average daytime BP, average nighttime, average 24-hour BP and overall ABPM at the ESH cutoffs for diagnosing hypertension on ABPM

| **24-hour ABPM parameters** | **People with HIV** **n (%)** | **People without HIV** **n (%)** |
| --- | --- | --- |
| Average daytime BP | 142 (29.4) | 152 (31.9) |
| Average nighttime BP | 236 (48.9) | 236 (49.6) |
| Average 24-hour BP | 167 (34.6) | 171 (35.9) |
| Overall ABPM | 260 (53.8) | 256 (53.8) |

Table S3: Hypertensive diagnostic phenotypes for uAOBP based on the ESH cutoffs for diagnosing hypertension. The table below summarizes the hypertensive diagnostic phenotypes when uAOBP is compared to average 24-hour BP at the ESH BP cutoffs for diagnosing hypertension. P values were generated from a chi square test comparing the distribution of participants’ hypertensive diagnostic phenotypes by HIV infection status. 

| **Hypertension diagnostic phenotype** | **People with HIV** **n (%)** | **People without HIV** **n (%)** | **Chi square p value** |
| --- | --- | --- | --- |
| Sustained normotension | 312 (64.6) | 295 (62.0) | 0.385 |
| White-coat hypertension | 4 (0.8) | 10 (2.1) |  |
| Masked hypertension | 124 (25.7) | 127 (26.7) |  |
| Sustained hypertension | 43 (8.9) | 44(9.2) |  |

Table S4: Hypertensive diagnostic phenotypes for uAOBP and hypertensive subgroups based on the ESH cutoffs for diagnosing hypertension. The table below summarizes the overall distribution of hypertensive subgroups (based on average asleep and awake readings on ABPM) for the four hypertensive diagnostic phenotypes when uAOBP is compared to average 24-hour BP at the ESH BP cutoffs for diagnosing hypertension. 

| **Hypertensive diagnostic phenotypes** | **Hypertensive subgroups** |  |  |  |
| --- | --- | --- | --- | --- |
|  | Isolated nocturnal hypertension n (%) | Isolated daytime hypertension n (%) | Ambulatory hypertension n (%) | Ambulatory normotension n (%) |
| Sustained normotension | 155 (25.5) | 11 (1.8) | 0 (0.0) | 441 (72.7) |
| Whitecoat hypertension | 9 (64.3) | 2 (14.3) | 1 (7.1) | 2 (14.23) |
| Masked hypertension | 55 (21.9) | 27 (10.8) | 167 (66.5) | 2 (0.8) |
| Sustained hypertension | 1 (1.2) | 2 (2.3) | 84 (96.6) | 0 (0.0) |

Stratified analyses of diagnostic performance at the ESH BP cutoffs

Table S5: Diagnostic performance of uAOBP compared to average 24-hour BP stratified by age and HIV infection status

| **Age category** | **Prevalence** **% (95% CI)** | **Sensitivity** **% (95% CI)** | **Specificity** **% (95% CI)** | **PPV** **% (95% CI)** | **NPV** **% (95% CI)** | **AUC** **% (95% CI)** |
| --- | --- | --- | --- | --- | --- | --- |
| **People without HIV** |  |  |  |  |  |  |
| 30 – 39 years | 22.0 (16.0, 29.1) | 40.5 (24.8, 57.9) | 92.4 (86.4, 96.3) | 60.0 (38.7, 78.9) | 84.6 (77.6, 90.1) | 0.67 (0.58, 0.75) |
| 40 – 49 years | 36.0 (29.0, 43.4) | 59.7 (47.0, 71.5) | 95.0 (89.3, 98.1) | 87.0 (73.7, 95.1) | 80.7 (73.2, 86.9) | 0.77 (0.71, 0.84) |
| 50 – 59 years | 55.0 (45.0, 65.8) | 58.8 (44.2, 72.4) | 78.0 (62.4, 89.4) | 76.9 (60.7, 88.9) | 60.4 (46.0, 73.5) | 0.68 (0.59, 0.78) |
| More than 60 years | 53.0 (34.0, 71.7) | 81.3 (54.4, 96.0) | 71.4 (41.9, 91.6) | 76.5 (50.1, 93.2) | 76.9 (46.2, 95.0) | 0.76 (0.61, 0.92) |
| **People with HIV** |  |  |  |  |  |  |
| 30 – 39 years | 23.0 (16.0, 31.4) | 42.9 (24.5, 62.8) | 97.9 (92.5, 99.7) | 85.7 (57.2, 98.2) | 85.2 (77.1, 91.3) | 0.71 (0.61, 0.80) |
| 40 – 49 years | 31.0 (25.0, 37.3) | 53.7 (41.1, 66.0) | 96.0 (91.6, 98.5) | 85.7 (71.5, 94.6) | 82.4 (75.9, 87.7) | 0.75 (0.69, 0.81) |
| 50 – 59 years | 48.0 (39.0, 57.6) | 50.0 (36.6, 63.4) | 95.2 (86.5, 99.0) | 90.6 (75.0, 98.0) | 67.0 (56.2, 76.7) | 0.73 (0.66, 0.80) |
| More than 60 years | 61.0 (39.0, 80.3) | 57.1 (28.9, 82.3) | 88.9 (51.8, 99.7) | 88.9 (51.8, 99.7) | 57.1 (28.9, 82.3) | 0.73 (0.56, 0.91) |

Table S6: Diagnostic performance of uAOBP compared to average 24-hour BP stratified by sex and HIV infection status. The table below summarizes the measures of diagnostic performance for uAOBP stratified by sex for people with and without HIV when compared to average 24-hour BP.

| **Diagnostic performance measure** | **People with HIV** |  | **People without HIV** |  |
| --- | --- | --- | --- | --- |
|  | **Male**  **n (95% CI)** | **Female** **n (95% CI)** | **Male** **n (95% CI)** | **Female** **n (95% CI)** |
| **Prevalence** | 47.0 (38.0, 55.4) | 29.0 (25.0, 34.6) | 46.0 (28.0, 54.7) | 31.0 (26.0, 36.6) |
| **Sensitivity** | 100 (95.3,100) | 31.0 (22.1, 41.0) | 13.2 (6.23, 23.6) | 34.0 (24.9, 44.0) |
| **Specificity** | 17.9 (9.61, 29.2) | 98.3 (95.8, 99.5) | 94.9 (87.5, 98.6) | 97.3 (94.3, 99.0) |
| **Positive predictive value** | 100 (73.5,100) | 88.6 (73.3, 96.8) | 69.2 (38.6, 90.9) | 85.4 (70.8, 94.4) |
| **Negative predictive value** | 58.0 (49.1, 66.6) | 77.4 (72.3, 82.0) | 56.0 (47.1, 64.5) | 76.4 (71.1, 81.2) |
| **AUC** | 0.59 (0.54, 0.64) | 0.65 (0.60, 0.69) | 0.54 (0.49, 0.59) | 0.66 (0.61, 0.70) |
| **Likelihood Ratio positive** | - | 18.6 (6.74, 51.3) | 2.61 (0.84, 8.11) | 12.8 (5.56, 29.5) |
| **Likelihood Ratio negative** | 0.82 (0.73,0.92) | 0.70 (0.62, 0.80) | 56.0 (47.1, 64.5) | 0.68 (0.59, 0.78) |

AUC – area under receiver operating characteristic curve

‘-’ Likelihood ratio positive cannot be calculated as the positive predictive value and specificity are 100%

**Multivariable logistic regression analysis of predictors of hypertension diagnosis** 

Table S7: Predictors of hypertension diagnosis by uAOBP. The table below summarizes the results of a multivariable logistic regression model evaluating the predictors of a higher likelihood of having a diagnosis of hypertension when BP is measured by uAOBP.

| **Sociodemographic characteristics** | **Odds Ratio (OR)** | **95% confidence interval** | **p-value** |
| --- | --- | --- | --- |
| **HIV infection status** |  |  |  |
| People with HIV | - |  |  |
| People without HIV | 1.04 | 0.78, 1.38 | 0.810 |
| **Age Group** |  |  |  |
| 30-39 years | - |  |  |
| 40-49 years | 1.40 | 1.01, 1.96 | 0.045 |
| 50-59 years | 2.56 | 1.70, 3.86 | <0.001 |
| More than 60 years | 4.72 | 2.10, 10.64 | <0.001 |
| **Sex** |  |  |  |
| Female | - |  |  |
| Male | 2.72 | 1.94, 3.81 | <0.001 |
| **Body mass index (BMI)** |  |  |  |
| Underweight | - |  |  |
| Healthy weight | 1.69 | 1.00, 2.84 | 0.049 |
| Overweight | 3.00 | 1.56, 5.75 | 0.001 |
| Obesity | 3.06 | 1.42, 6.59 | 0.004 |

Supplementary analyses of diagnostic performance using the ACC/AHA guidelines for diagnosing hypertension

Table S8: Prevalence of hypertension based on 24- hour ABPM parameters at the ACC/AHA BP cutoffs for diagnosing hypertension

| **24-hour ABPM parameters** | **People without HIV** **n (%)** | **People with HIV** **n (%)** |
| --- | --- | --- |
| Average daytime BP | 244 (51.3) | 227 (47.0) |
| Average nighttime BP | 360 (75.6) | 355 (73.5) |
| Average 24-hour BP | 296 (62.2) | 269 (55.7) |
| Overall ABPM | 375 (78.8) | 370 (76.6) |

Table S9: Hypertensive diagnostic phenotypes for uAOBP based on the ACC/AHA cutoffs for diagnosing hypertension. The table below summarizes the hypertensive diagnostic phenotypes when uAOBP is compared to average 24-hour BP at the ACC/AHA recommended BP cutoffs. P values were generated from a chi square test comparing the distribution of participants’ hypertensive diagnostic phenotypes by HIV infection status. 

| **Hypertension diagnostic phenotype** | **People with HIV** **n (%)** | **People without HIV** **n (%)** | **Chi square p value** |
| --- | --- | --- | --- |
| Normotensive | 173 (36.3) | 212 (43.9) | 0.022 |
| White Coat Hypertension | 7 (1.5) | 2 (0.4) |  |
| Masked hypertension | 176 (37.0) | 174 (36.0) |  |
| Sustained hypertension | 120 (25.2) | 95 (19.7) |  |

Table S10: Hypertensive groups based on average 24-hour BP at the ACC/AHA cutoffs for diagnosing hypertension. The table below summarizes the hypertensive groups based on when BP is above ACC/AHA cutoffs for diagnosing hypertension on average 24-hour BP. P values were generated from a chi square test comparing the distribution of participants’ hypertensive groups by HIV infection status. 

| **Hypertension diagnostic phenotype** | **People with HIV** **n (%)** | **People without HIV** **n (%)** | **Chi square p value** |
| --- | --- | --- | --- |
| ^* ^Ambulatory normotension | 114 (23.6) | 102 (21.4) | 0.615 |
| Isolated daytime hypertension | 14 (2.9) | 14 (2.9) |  |
| Isolated nocturnal hypertension | 142 (29.4) | 130 (27.3) |  |
| ^*^ Ambulatory hypertension | 213 (44.1) | 230 (48.3) |  |

^* ^Ambulatory normotension refers to sustained daytime and nighttime normotension while ambulatory hypertension refers to sustained daytime and nighttime hypertension on average 24-hour BP

Table S11: Diagnostic performance of uAOBP among included participants by HIV infection status at the ACC/AHA cutoffs for diagnosing hypertension. This table summarizes the measures of diagnostic performance for uAOBP when compared to average 24-hour BP on ABPM

| **Diagnostic performance measure** | **Participant groups by HIV infection status** **n (95% CI)** |  |
| --- | --- | --- |
|  | **People with HIV** | **People without HIV** |
| **Prevalence** | 56.0 (51.0, 60.2) | 62.0 (58.0, 66.6) |
| **Sensitivity** | 35.3 (29.6, 41.4) | 40.5 (34.9, 46.4) |
| **Specificity** | 99.1 (96.7, 99.9) | 96.1 (92.2, 98.4) |
| **Positive predictive value** | 97.9 (92.7, 99.7) | 94.5 (89.0, 97.8) |
| **Negative predictive value** | 54.9 (49.8, 60.0) | 49.6 (44.2, 54.9) |
| **AUC** | 0.67 (0.64, 0.70) | 0.68 (0.65, 0.72) |
| **Likelihood Ratio positive** | 37.8 (9.42, 152) | 10.4 (4.98, 21.8) |
| **Likelihood Ratio negative** | 0.65 (0.60, 0.71) | 0.62 (0.56, 0.68) |

AUC – area under receiver operating characteristic curve

Supplementary analysis of diagnostic performance of uAOBP average daytime BP as the reference standard

Table S12: Hypertensive diagnostic phenotypes for uAOBP when compared to average daytime BP at the ESH cutoffs for diagnosing hypertension. 

| **Hypertension diagnostic phenotype** | **People with HIV** **n (%)** | **People without HIV** **n (%)** | **Chi square p value*** |
| --- | --- | --- | --- |
| Normotensive | 337 (69.8) | 316 (66.4) | 0.513 |
| White Coat Hypertension | 4 (0.8) | 8 (1.2) |  |
| Masked hypertension | 99 (20.5) | 106 (22.3) |  |
| Sustained hypertension | 43 (8.9) | 46 (9.7) |  |

* P values were generated from a chi square test comparing the distribution of participants’ hypertensive diagnostic phenotypes by HIV infection status 

Table S13: Diagnostic performance of uAOBP among included participants by HIV infection status using average daytime BP as the reference standard at the ESH cutoffs for diagnosing hypertension. 

| **Diagnostic performance measure** | **Participant groups by HIV infection status** **n (95% CI)** |  |
| --- | --- | --- |
|  | **People with HIV** | **People without HIV** |
| **Prevalence** | 29.0 (25.0, 33.7) | 32.0 (28.0, 36.3) |
| **Sensitivity** | 30.3 (22.9, 38.5) | 30.3 (23.1, 38.2) |
| **Specificity** | 98.8 (97.0, 99.7) | 97.5 (95.2, 98.9) |
| **Positive predictive value** | 91.5 (79.6, 97.6) | 85.2 (72.9, 93.4) |
| **Negative predictive value** | 77.3 (73.1, 81.1) | 74.9 (70.5, 79.0) |
| **AUC** | 0.65 (0.61, 0.68) | 0.64 (0.60, 0.68) |
| **Likelihood Ratio positive** | 25.8 (9.44, 70.6) | 12.3 (5.93, 25.3) |
| **Likelihood Ratio negative** | 0.71 (0.63, 0.79) | 0.72 (0.64, 0.80) |

AUC – area under receiver operating characteristic curve

**Figure S1: Correlation of A. systolic and B. diastolic uAOBP measurements and average daytime BP on ABPM**

Figure S2: Bland Altman plots of level of agreement for A. systolic and B. diastolic uAOBP and average daytime BP on ABPM for people without HIV. 

| **A.** |
| --- |
|  |
| **B.** |
|  |

The correlation coefficients (Pitman test) between the difference and mean were r=−0.372, P= <0.001 for systolic measurements and r=-0.247, P= <0.001 for diastolic measurements.

Figure S3: Bland Altman plots of level of agreement for A. systolic and B. diastolic uAOBP and average daytime BP on ABPM for people with HIV. 

| **A.** |
| --- |
|  |
| **B.** |
|  |

The correlation coefficients (Pitman test) between the difference and mean were r=−0.428, P= <0.001 for systolic measurements and r=-0.277, P= <0.001 for diastolic measurements.
